# Forgotten Gangliosides: *O*-Acetylated and Lactone Gangliosides

**DOI:** 10.3390/ijms27073188

**Published:** 2026-03-31

**Authors:** Laura Mauri, Stefano Vaghi, Sandro Sonnino

**Affiliations:** Department of Medical Biotechnology and Translational Medicine, University of Milan, 20054 Segrate, Italy; laura.mauri@unimi.it (L.M.); stefano.vaghi@unimi.it (S.V.)

**Keywords:** gangliosides, alkali-labile gangliosides, *O*-acetyl-gangliosides, ganglioside lactones

## Abstract

Gangliosides containing *O*-acetyl-sialic acid or a sialic acid forming a lactone ring, classified as alkali-labile gangliosides (ALGs), were the focus of extensive research until the late 1990s. Their identification and isolation for structural characterization require strict avoidance of alkaline treatments, a common step in standard ganglioside purification protocols used to eliminate glycerophospholipid contamination. After 2000, scientific interest in alkali-labile gangliosides declined significantly, resulting in a dearth of new information and few updates on their biological significance. However, in recent years, new insights have emerged regarding the potential of anti-acetylated ganglioside antibodies for specific tumor treatments and how ganglioside structures can modulate the activity of membrane receptors through specific interactions. Further knowledge of alkali-labile gangliosides could be very useful for better understanding their significance in both normal and tumor cells. In this manuscript, we intend to revisit the existing knowledge on alkali-labile gangliosides and form hypotheses regarding their possible roles in cells.

## 1. General Introduction to Gangliosides

**Figure 1:**
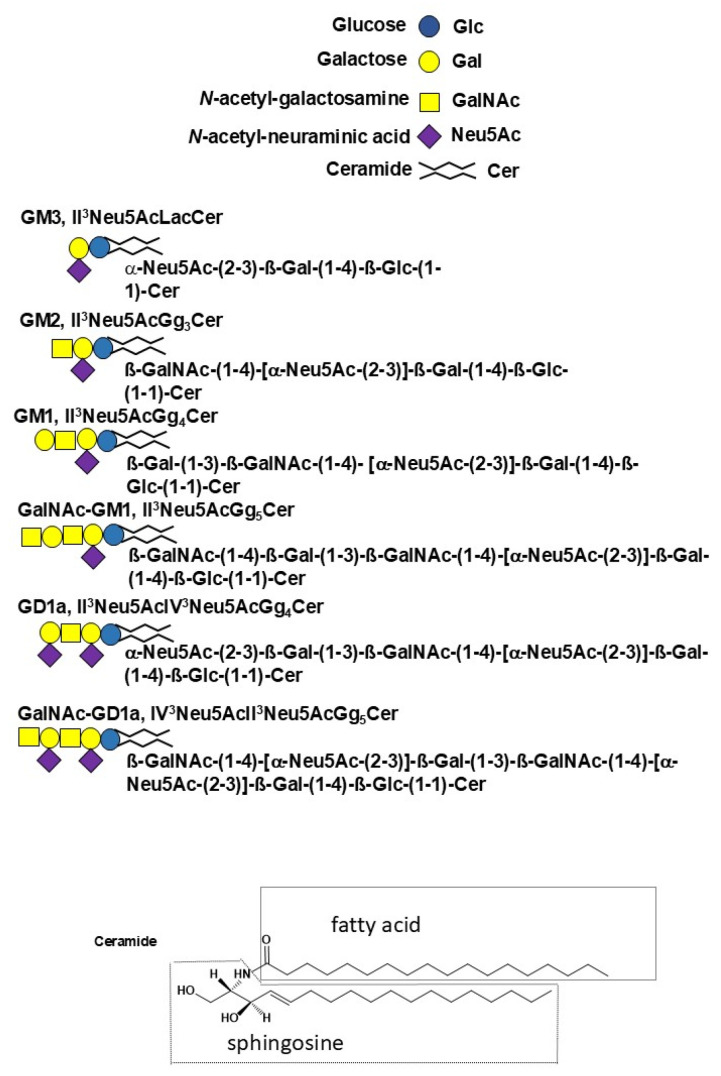
Schematic representation of gangliosides. The saccharide residues are represented using their official color and geometrical shape. The structure of ceramide is used as an example. In gangliosides, the structure of ceramide is heterogeneous. In brain gangliosides, both C18- and C20- sphingosine are present, and stearic acid accounts for over 90% of the total fatty acids. In extra-nervous-system tissues, C18-sphingosine is the long-chain base, occasionally with traces of the other species; fatty acids are very variable, with 14 to 24 carbons, with or without double bonds, and with or without hydroxyl groups (in the alpha position).

Gangliosides are sialic-acid-containing glycosphingolipids. They are ubiquitous components of vertebrate plasma membranes and particularly abundant in the nervous system. Gangliosides contain up to six sialic acid residues linked to the linear neutral oligosaccharide chain either as single residues or as di- or tri-sialosyl chains. Gangliosides are amphiphilic compounds containing a lipid moiety called ceramide linked to an oligosaccharide chain (for a schematic representation of a ganglioside with conventional color and geometric symbols for carbohydrates, see Figure 1). Considering the gangliosides of all tissues, including nervous, extra-nervous, and tumor tissues, the structure of ganglioside is extremely variable both in terms of the oligosaccharide hydrophilic chain and the ceramide structure. The oligosaccharide chains differ in sugar composition, sugar sequence, glycosidic linkage anomericity, and glycosidic linkage position. The ceramide component also shows remarkable structural heterogeneity due to both the fatty acid and log chain base (the latter commonly called sphingosine), which can vary in terms of length, unsaturation, hydroxylation, and, in some cases, methylation. Although many gangliosides have been identified, most of them occur only as minor or trace species. In contrast, a few, such as GM1, GD1a, GD1b, and GT1b, are abundant. Gangliosides characteristic of the nervous system (where gangliosides are abundant) and those associated with tumor tissues (although present in minimal amounts) have been studied in detail, and important findings are available in the literature. In contrast, our knowledge of the majority of minor gangliosides remains limited, primarily due to analytical challenges encountered in their isolation and structural identification.

The glycosidic linkages connecting the sugars within the oligosaccharide chain are stable under alkaline conditions. Thus, gangliosides are considered alkali-stable compounds, unless they are exposed to very drastic conditions, which can induce deacetylation of *N*-acetyl-amino sugars. Nevertheless, a group of minor gangliosides that proved to be unstable when subjected to alkaline treatments (known as alkali-labile gangliosides, ALGs) has attracted considerable scientific interest. Numerous researchers have investigated these molecules, publishing several studies on their properties until the late 1990s.

Unexpectedly, since that period, only a few articles on alkali-labile gangliosides have been published.

**Figure 2:**
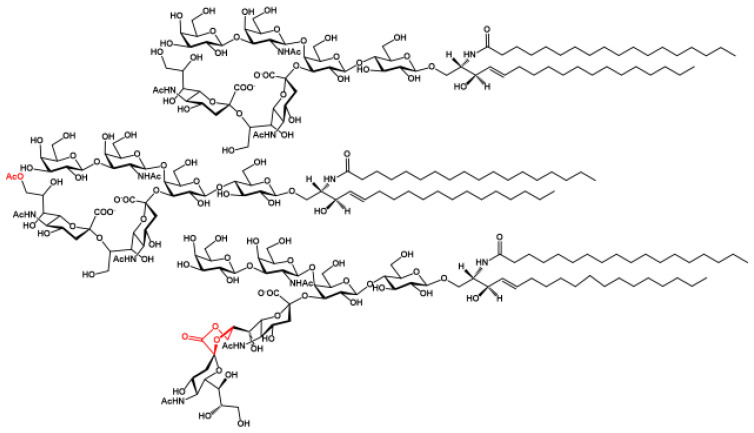
The flat structures of the gangliosides GD1b, *O*-acetyl-GD1b, and GD1b-lactone (from top to bottom) are schematically represented to show Neu5Ac, Neu5,9Ac_2_, and the Neu5Ac-(2-8,1-9)-Neu5Ac lactone ring. Torsional angles of the sugar linkages are only indicative; correct tridimensional representations of the GD1b and GD1b-lactone central oligosaccharide sequences are reported in Figure 3 according to reference [2]. In gangliosides, only Neu5,9Ac_2_ has been found. Other positions of sialic acid exist in *O*-acetylated glycoproteins. Only the external sialic acid of GD1b has been found *O*-acetylated, but the possibility that a different structure exists in nature cannot be excluded. In GD1b–lactone (and in GD3-lactone, which lacks the external disaccharide with respect to GD1b), only the lactone ring Neu5Ac-(2-8,1-9)-Neu5Ac has been found. The lactone ring Neu5Ac-(2-3,1-2)-Gal has been found in the GM3 lactone.

Alkali-labile gangliosides, ALGs, contain *O*-acety-sialic acid or lactonized sialic acid [1] (see Figure 2). After treatment with sodium hydroxide, both *O*-acetyl-gangliosides and lactonized gangliosides revert to the stable compound. In contrast, lactonized gangliosides treated with ammonia partially revert to the stable compound and are partially converted into the amide derivative. Alkali-labile gangliosides are “vanishing” molecules because the procedures commonly used for their preparation typically involve alkaline treatments used to remove contaminating glycerophospholipids, conditions under which these gangliosides are degraded.

**Figure 3:**
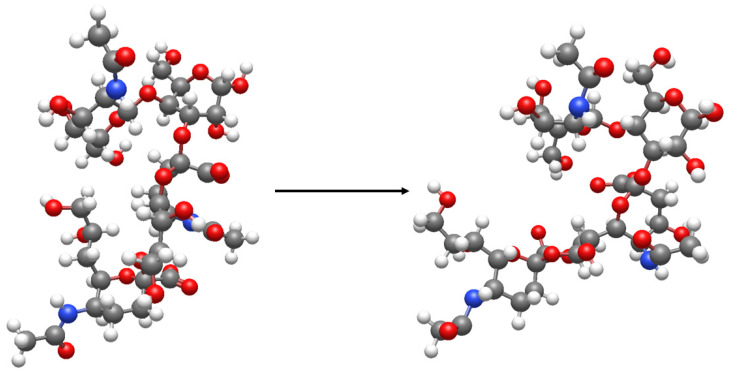
Representation of the changes in conformation that occur when moving from GD1b to GD1b lactone. In GD1b, the external sialic acid interacts with *N*-acetylgalactosamine. But this no longer occurs after lactonization.

Nevertheless, when alkaline conditions are avoided, ALGs can be detected in the total ganglioside mixture extracted from the brains of humans, rodents (including rats), birds, rabbits, horses, pigs, cows, sheep, cats, freshwater lampreys, other fish, and reptiles [1,3,4,5,6,7] in concentrations ranging from a few percent to up to 50% of the total sialic acid content, and they can also be found in tumor tissues [8,9,10,11,12].

So far, most of the results have been analytical and structural and provide little insight into the biological significance of these gangliosides’ alkali-labile properties and the roles they play in membrane organization and cell physiology. However, the presence of ALGs in nearly all brains analyzed thus far [1], their lack of synthesis during hibernation or acclimatization in some animals [4,13,14], their resistance to the enzyme sialidase, the change in their content throughout brain development [15], and their occurrence in tumor tissues [8,9,10,11,12] could suggest they play specific roles in both physiological and pathological processes.

Both *O*-acetylation and lactonization reduce the hydrophilicity of sialic acid, thereby altering the amphiphilic equilibrium between the oligosaccharide chain and the ceramide moiety. Gangliosides’ structural features are considered to be the driving forces for the formation of membrane micro-domains [16], characterized by high concentrations of dipalmitoylphosphatidylcholine, sphingolipids, and cholesterol. Therefore, ALGs may play an important role in modulating membrane organization and ganglioside–protein interactions required for ganglioside-dependent cellular signaling [17].

## 2. Akali-Labile Gangliosides, ALGs

### 2.1. The Structures of O-Acetyl-Gangliosides

**Figure 4:**
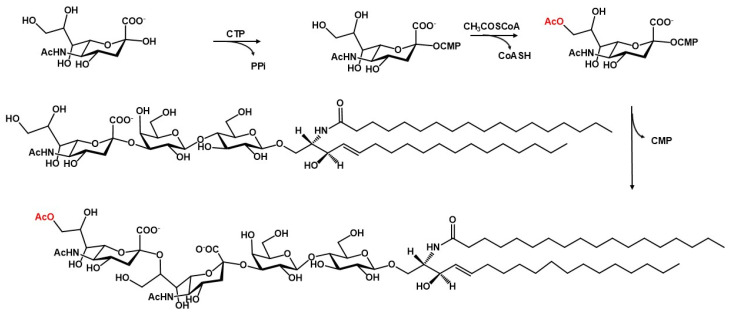
Scheme of the biosynthesis of an *O*-acetyl-ganglioside. As an example, the synthesis of *O*-acetyl-GD3 is shown.

1. *O*-Acetylated gangliosides are acetylated on the sialic acid. This modification requires a specific sialic acid *O*-acetyl-transferase and a CMP-sialic acid as a donor substrate [18,19] (see Figure 4). The enzyme exists in both membrane and cytosolic forms. In humans, the membrane-associated form CASD1 (capsule structure1 domain-containing 1) localizes to the Golgi apparatus and is active on sialic acid and both gangliosides and sialoproteins.

In the human Golgi system, sialic acid *O*-acetylation involves the activity of sialic acid *O*-acetyltransferases (SOATs) and sialic acid esterases (SIAEs) that add and remove *O*-acetyl groups on sialic acid, respectively [19]. In the Golgi system, *O*-acetylation of sialic acid occurs on the free CMP-activated sialic acid and takes place before sialyltransferase incorporates the sialic acid into a glycosphingolipid or glycoprotein. However, *O*-acetylation may also occur after sialylation [19]. The balance between the activity of the sialic acid *O*-acetyl transferase and that of the sialic acid acetyl esterase determines the final amount of *O*-acetyl-sialic acid. Both *N*-acetyl-neuraminic acid (Neu5Ac) and *N*-glycolyl-neuraminic acid (Neu5Gc), as free sugars or inserted in the glycoconjugate, serve as substrates for the enzyme, transferring an acetyl group to position 4, 7, 8, or 9 of the sialic acid molecule. Nevertheless, it was recently suggested that CASD1 has a preference for position 9 [20]. After the initial modification, the acetyl group undergoes intramolecular migration, typically shifting from positions 7 and 8 to position 9. The main *O*-acetylated gangliosides contain *N*-acetyl-neuraminic acid that has been *O*-acetylated in position 9, whose official abbreviation is Neu5,9Ac_2_.

### 2.2. The Structures of Ganglioside Lactones

Lactones are often unstable compounds, and their identification requires great care. Nevertheless, at the end of the 1960s, the existence of ganglioside lactones was hypothesized [21,22], and they were later proposed to occur in ganglioside mixtures extracted from rodent brains [23]. Gangliosides containing a disialosyl chain linked at position 3 of the galactose bonded to glucose, such as GD1b and GD3, appear to be more suitable for lactonization. A lactone derived from GD1b in which the carboxyl group of the external *N*-acetyl-neuraminic acid forms a lactone ring with the -OH group at position 9 of the inner *N*-acetyl-neuraminic acid was isolated from human brains [24].

A ganglioside lactonase has not yet been isolated; however, several observations suggest its existence is possible: 1. Cultured cerebellar granule cells contain GD1b-lactone, whose levels increase when cells are administered GD1b; conversely, astrocytes, which do not contain GD1b-lactone, still do not produce it even after GD1b administration [25]. 2. When GD1b is injected into the rat brain, it is partially converted into GD1b-lactone in a time-dependent manner [26]. 3. In the human brain, GD1b-lactone content increases with age [24].

Nevertheless, it is important to note that GD1b and GD1b lactone rapidly reach an equilibrium under acidic conditions. This suggests that a specific cellular chemical process, or alternative to the enzymatic process, might be responsible for ganglioside lactonization [27].

## 3. Discussion and Hypothesis

Gangliosides are components of the plasma membranes of vertebrates, and they are highly abundant in the nervous system. The patterns and neuronal content of gangliosides vary during brain development and show species-differing patterns at the end of brain development. In contrast, the final ganglioside content per mg of brain protein or per mg of brain weight does not differ substantially across species. A portion of the total ganglioside content in the brain is alkali-labile, and its relative amount decreased markedly over the course of evolution from fish to mammals, changing from accounting for over 50% to only a small percent of the total brain ganglioside pool [1,3,4,5,6,7]. Moreover, in species where it has been examined, levels of ALG correlate with the environmental temperature in which each organism lives [13], and they increase progressively during brain development, together with the alkali-stable gangliosides [15]. These observations support the hypothesis that ALG is linked to neural features essential for sustaining species-specific physiological stability and optimal function. Of course, factors such as intelligence, the ability to form increasingly complex neural networks, the ability to store information, and the level of neural plasticity should be considered when attempting to explain or understand ALGs’ behavior. Unfortunately, it is difficult to reach a conclusion since we have limited information, and our suggestions remain a hypothesis. Nevertheless, the evidence allows us to state, with some confidence, that a link must exist between ALGs and some cellular properties.

If we compare geographical areas far from the equator with those close to it, it becomes evident that environmental temperatures can be very different. Humans face this issue by wearing appropriate clothing, using internal heating or cooling in indoor environments, and taking proper care of their animals. However, wild animals cannot regulate their environments, and it appears that one of the strategies they employ to cope with thermal conditions may be associated with the ALG content of the brain. For instance, among fish species, Antarctic fish and cold-acclimated fish exhibit ALG levels representing 53–67% and 45–51% of their total brain ganglioside content, respectively. These values are higher than those found in tropical or heat-acclimated fish, which show levels around 35–40% [13].

In addition, it is known that some rodents modify their brain ALG content in response to environmental temperature changes that occur with seasonal cycles.

The brains of active dormice (*Glis glis*) are rich in ALGs in all areas of the central nervous system, displaying a minimum of 10.2% in the olfactory bulb and a maximum of 30.1% in the spinal cord in terms of total ganglioside content. The main ALGs in all areas are *O*-acetyl-GT1b and *O*-acetyl-GQ1b [14] However, ALGs are absent in the brains of hibernating animals, being present in only trace amounts in the brain stem and olfactory bulb.

On the other hand, ALG synthesis also appears to follow different changes as a function of environmental temperature, even when considering other rodents. In hamsters, for example, brain ALG levels show an opposite pattern compared to those in dormice: active hamsters display about 4% ALG, whereas “torpid” (non-hibernating but cold-adapted) hamsters show levels of approximately 9%.

The above information indicates that the brain’s synthesis processes vary according to environmental conditions, whether natural or experimentally imposed.

However, other conditions, such as nervous system development, also involve changes in ALG synthesis. In this regard, it is known that ALG levels progressively increase throughout the development of the rabbit nervous system, with particularly notable increases in levels of *O*-acetyl GT1b and *O*-acetyl GQ1b. These increases are especially pronounced in the cerebellum. *O*-acetyl-GD3 is present in the later stage of rat embryonic development and the early postnatal period [28], wherein it could play an opposite role to GD3, showing anti-apoptotic properties [29]. The situation in cancer tissues from cancers such as breast cancer, basalioma, tumors of neuroectodermal origin, childhood lymphoblastic leukemia, and glioblastoma [29] seems quite different: here, the aberrant glycosylation processes lead to a high *O*-acetyl-GD3 content.

The role of gangliosides in the organization and function of neuronal plasma membranes has been widely discussed. Unsurprisingly, ganglioside content varies according to the specific activity of neurons, which must provide specific signals necessary to regulate cellular metabolism in response to external temperatures or other conditions, such as brain development.

Gangliosides, due to their structures, play a crucial role in membrane organization and membrane functions [16]. At the water–lipid interface, the oligosaccharide chains form reciprocal hydrogen bonds between the sugar hydroxyl groups and the oligosaccharide surrounding water molecules. In addition to this, the amide group of ceramide is a rigid group comprising six atoms in a planar conformation. The group has a perpendicular orientation towards the axes of the two hydrocarbon chains, whose parallel orientation is stabilized by the presence of a double bond at position 4–5 of sphingosine. The characteristic presence of the hydroxyl group at position 2, the amide bond, and the carbonyl oxygen allows sphingolipids to form hydrogen bonds, acting simultaneously as hydrogen bond donors and acceptors [30]. Therefore, these features are conducive to a stable network of interactions among sphingolipids and neighboring molecules, including dipalmitoylphosphatidylcholine, which is enriched in lipid rafts [31]. These interactions thermodynamically favor the aggregation and stabilization of membrane lipid domains, known as lipid rafts [16]. Lipid rafts are also rich in cholesterol, sphingomyelin, and other sphingolipids, which contribute to the formation of highly ordered and rigid regions within the plasma membrane. Lipid rafts contain only a minor quantity of proteins, typically not exceeding 3–5% of the total membrane protein content. These proteins are receptors and enzymes (often kinases), whose activity is dependent on and regulated by gangliosides through specific interactions [32,33,34]. The acetylation of sialic acid reduces the polarity of gangliosides, whereas sialic acid’s lactonization further decreases their polarity and greatly increases molecular rigidity. This effect arises from significant conformational changes and altered interactions between sialic acid and *N*-acetyl-galactosamine (see Figure 3). Thus, ALGs exhibit very different properties from their parent compounds. Unfortunately, no information is currently available regarding ALG–protein interactions. However, it is known that even minor changes in gangliosides’ oligosaccharide structures prevent them from being able to regulate protein functions. The interaction between the oligosaccharide portion of GM1 and the TrkA receptor has been studied in detail. This interaction is highly specific and necessary for the activity of the receptor after the release of NGF from the cell. Even minor differences in the oligosaccharide structures of gangliosides render the TrkA receptor inactive and block TrkA’s cellular signal [33]. We know that artificial changes to the lipid-raft composition immediately lead to a general lipid rearrangement [35], and this could lead to different ganglioside–protein interactions. Unfortunately, no data are currently available regarding the behavior of other lipid-raft components in the presence of ALGs. Changes in the lipid-raft content of cholesterol and/or sphingomyelin associated with ALGs could contribute to the emergence of new membrane properties.

If *O*-acetylated gangliosides are considered elusive compounds that will disappear unless great care is taken to avoid alkaline treatments, lactone gangliosides can be considered real ghosts.

Gangliosides are acidic compounds due to the pKa of sialic acid, which is around 2.2–2.5. This makes the oligosaccharides in gangliosides very hydrophilic and allows interactions with surrounding water. Dozens of water molecules surround the oligosaccharide chain, forming hydrogen bonds with its hydroxyl groups [36]. In addition, gangliosides interact with positive ions—which are required to balance their negative charge and reduce electronegativity repulsion—as well as with adjacent membrane molecules. Upon lactonization, gangliosides lose their negative charge, reducing membrane electronegativity. It is known that the activity of two membrane enzymes, sialyltransferase [37] and the sialidase Neu3 [2], monitor the number of sialic acid residues. Therefore, lactonization of gangliosides is an alternative mechanism for the determination of ganglioside-dependent membrane electronegativity. Furthermore, ganglioside lactones containing a disialosyl residue, such as GD1b and GD3, are sialidase-resistant [24], thus preventing enzymatic modification of the structure of ganglioside on its surface. Also important are the conformational changes induced by lactonization, which result from substantial alterations in the pre-existing glycosidic torsional angles (see Figure 3) [38]. This contributes to the production of gangliosides, as well as the membranes in which they are inserted, which are very rigid. Consequently, it would not be surprising to find that several ganglioside receptor–protein interactions either occur differently or fail to occur altogether as a result of the lactonization process.

As noted above, although the existence of a ganglioside lactonase has been suggested, the enzyme has never been isolated. However, it is important to recall that lactonization of gangliosides occurs spontaneously under acidic conditions. Indeed, at an H^+^/GD1b ratio of 1, 50% of GD1b is lactonized, and for higher H^+^/GD1b ratios (up to 7), the GD1b-to-GD1b-lactone ratio reaches a maximum value of 3:7 [27]. Changes in membrane pH toward more acidic values represent an important process, one necessary for the activity of membrane-associated enzymes resulting from the fusion of lysosomes with plasma membranes [39]. These enzymes show activity at an acidic pH, which, on the cell surface, is achieved through the activity of the Na^+^/H^+^ antiporter. Neurons are enriched in this Na^+^/H^+^ antiporter, which is a component of the same lipid raft as gangliosides [40] and is under the control of kinases via phosphorylation. GD1b activates the phosphorylation cascade [41], and this rapidly acidifies the ganglioside microenvironment by exchanging extracellular Na^+^ for cytosolic H^+^. Under these conditions, the GD1b/GD1b-lactone equilibrium shifts strongly toward the lactone form, which, unlike GD1b, cannot activate kinase. At this point, the acidification process stops, Na^+^ is transported back to the extracellular space, and GD1b-lactone is converted back into GD1b.

**Figure 5:**
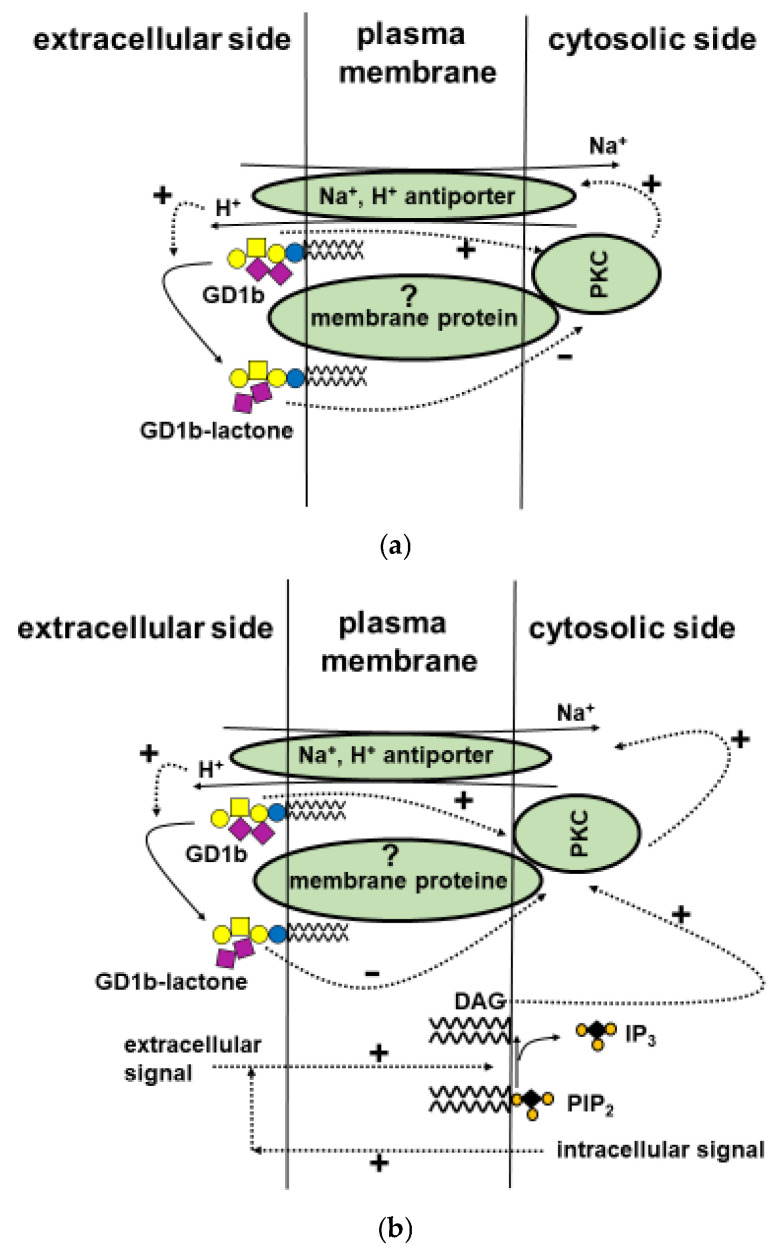
(**a**) Proposed process involving the GD1b↔GD1b-lactone equilibrium for modulation of membrane kinase activity. Gangliosides and Na^+^/H^+^ neuronal antiport are components of the same membrane microenvironment. Protein kinase-C, PKC, is under GD1b control and, when necessary, increases the effect of diacylglycerol. PKC is an activator of the H^+^/Na^+^ antiport that exchanges cytosolic protons with extracellular sodium ions. The pH of the plasma membrane becomes acidic, and this shifts the GD1b/GD1b-lactone equilibrium towards the GD1b-lactone end, resulting in a loss of the negative charge and a change in conformation. The GD1b-lactone blocks PKC activity, and this likely requires intervention of a membrane protein to connect the outer and inner two faces of the membrane where the ganglioside-lactone and PKC, respectively, are associated. Immediately, the sodium ions return to the outside and restore the correct cytosolic pH. Dotted arrows: Positive or negative regulations. Continuous arrows: Reactions. (**b**) Information is the same as in Figure 5 upper part, but in combination with PIP2 regulation.

The GD1b/GD1b-lactone equilibrium can regulate kinase activity on its own (Figure 5a) or act together with PIP2-dependent regulation, which can be triggered by both intra- and extra-cellular stimuli (Figure 5b).

There is currently no information explaining how the positive activity of the protein kinase depends on GD1b and disappears when GD1b is converted into its lactone form. It is worth recalling that both GD1b and GD1b-lactone extend the oligosaccharide chain from the outer layer of the plasma membrane, whereas the kinase is associated with the inner layer. Gangliosides could interact directly, albeit in a different way, with a transmembrane protein being capable of translating the regulatory signal to the kinase. Alternatively, they could modify lipid-raft organization because of the conformational changes induced by lactonization, which is responsible for the kinase’s shape and properties.

Cell membranes are structures that preserve cellular integrity while enabling continuous interaction between the intracellular and extracellular environments. Just as importantly, cell membranes can regulate important cellular functions by transmitting crucial information from the outside to the inside. Membrane composition and organization play key roles in these processes, which rely on the formation of micro-domains shaped by the particular physical and chemical characteristics of gangliosides.

Membranes are very dynamic structures whose organization is associated with their composition. Gangliosides, with their multiple structures generated at different stages of cellular life, during which different biosynthetic activities occur, contribute to the formation of specialized membrane domains. These domains facilitate interactions between gangliosides and membrane receptors or enzymes, thereby modulating their activity. Such regulatory mechanisms are essential for proper nervous system function [32]. Both the saccharide and ceramide portions play important roles in membrane organization and the subsequent interaction processes between gangliosides and proteins. Although information on the role of ALGs in membrane organization is not yet available, some principles are well-established. At a fixed oligosaccharide composition, gangliosides exhibit lateral phase separation to an extent dependent upon the length and unsaturation differences between the ganglioside long-chain base and phosphatidylcholine acyl chains [42]. Conversely, when the structure of ceramide remains constant, the extent of phase separation is dependent upon the number of sugar units present in the glycolipid. The presence of Ca^++^ enhances phase separation depending on the number of sialic acid residues, i.e., the number of negative charges [42]. Synthesis of ALG leads to a reduction in negative charges and, in some cases, a reduction in interactions with Ca^++^. It also consistently results in reduced hydrophilicity.

In addition to all the information above, we would like the reader to recall that ALGs could be a starting point for new therapies aimed at controlling cancer. Recent papers suggest, for example, that mimicry of GM3-lactone allows greater inhibition of migration and invasiveness of human melanoma [43], and anti-*O*-acetylated GD2 antibodies could be, alone or in combination with other drugs, new tools for fighting cancer associated with ganglioside metabolism [44,45].

Unfortunately, interest in ALGs, perhaps due to the analytical challenges associated with their detection and characterization, has progressively declined. Therefore, their contribution to cellular functions remains almost entirely unknown.

Alkali-labile gangliosides therefore persist as largely overlooked and forgotten compounds.

## Data Availability

No new data were created or analyzed in this study. Data sharing is not applicable.
